# A Bioeconomically Valuable Essential Oil from *Baccharis sinuata* Kunth in Southern Ecuador: Chemical Composition and Enantiomeric Profile

**DOI:** 10.3390/plants14193110

**Published:** 2025-10-09

**Authors:** Gianluca Gilardoni, Bryan Flores, Nixon Cumbicus, Omar Malagón

**Affiliations:** 1 Departamento de Química, Universidad Técnica Particular de Loja (UTPL), Calle Paris s/n y Praga, Loja 110107, Ecuador; ggilardoni@utpl.edu.ec; 2 Carrera de Bioquímica y Farmacia, Universidad Técnica Particular de Loja (UTPL), Calle Paris s/n y Praga, Loja 110107, Ecuador; beflores6@utpl.edu.ec; 3 Departamento de Ciencias Biológicas y Agropecuarias, Universidad Técnica Particular de Loja (UTPL), Calle Paris s/n y Praga, Loja 110107, Ecuador; nlcumbicus@utpl.edu.ec

**Keywords:** Asteraceae, chiral separation, mass spectrometry, (*R*)-(+)-limonene, δ-cadinene

## Abstract

The present research describes the chemical composition and the enantiomeric profile of a spicy green aroma essential oil, distilled from the dry leaves of *Baccharis sinuata* Kunth (Asteraceae). The distillation yield was as high as 3.0% by weight. The chemical analysis was conducted on two columns, coated with stationary phases of different polarity (5% phenyl—95% methyl polysiloxane, expressed by weight, and 100% polyethylene glycol). Major components (≥2.0% as an average value between the two columns) were as follows: β-pinene (4.9%), limonene (39.0%), (E)-β-caryophyllene (2.0%), bicyclogermacrene (2.7%), γ-cadinene (4.0%), δ-cadinene (7.3%), β-eudesmol (2.0%), α-eudesmol (3.0%), and α-cadinol (2.0%). For the enantioselective analysis, 10 enantiomeric pairs were investigated, using two capillary columns coated with different chiral selectors. As a result, (1*R*,5*R*)-(−)-α-thujene, (1*S*,5*S*)-(−)-α-pinene, and (1*R*,2*S*,6*S*,7*S*,8*S*)-(−)-α-copaene were enantiomerically pure, whereas (*R*)-(+)-limonene presented a 90.0% enantiomeric excess. All the other analysed chiral compounds were scalemic mixtures. The high distillation yield, its aroma, and the bibliographic bioactivity profile make this essential oil potentially interesting from a commercial point of view. To the best of the authors’ knowledge, this is the first description of an essential oil distilled from leaves of *B. sinuata*.

## 1. Introduction

**Figure 1:**
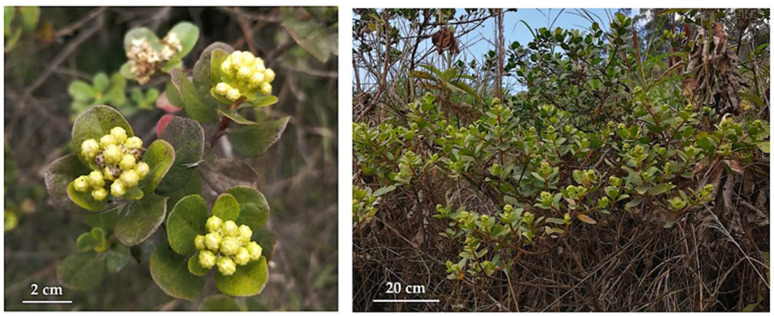
A specimen of *Baccharis sinuata* Kunth at the collection site (Photos by Gianluca Gilardoni).

Since ancient times, plants have been a major source of aromas and bioactive products for humans. Still today, aromatic plants and flowers constitute the raw material for natural flavour and fragrances, mainly in the form of essential oils (EOs) or their derivatives. On the other hand, medicinal plants still represent the principal drugs for millions of people around the world, and they continue to inspire medicinal chemists in search of new pharmaceutical active principles [1]. However, the phytochemistry of most European and many North American botanical species have been exhaustively studied, obliging chemists to focus on the flora of so called “megadiverse” countries. Among them, Ecuador must be mentioned [2]. For these reasons, our group has been studying Ecuadorian biodiversity for more than 20 years, discovering specialised metabolites from previously unstudied species [3]. Currently, we mainly focus on volatile fractions, describing the chemical composition, enantiomeric profile, biological activities, and olfactory properties of new EOs [4,5,6,7]. Natural products in general and EOs in particular are commercially important items that constitute an opportunity for the economy of rural communities, especially in developing countries. As a consequence, the description of new bioeconomically viable products usually generates an interest in preserving the vegetal species that act as raw materials, together with the ecological medium where they grow. Starting from this premise and with the aim of contributing to the preservation of the Ecuadorian biodiversity through biochemical knowledge, *Baccharis sinuata* Kunth was selected as a source of an unprecedented bioeconomically promising EO (see Figure 1). This species, belonging to the family Asteraceae, has been described in botanical libraries as endemic to Peru, growing between 2400 and 3800 m above the sea level [8,9]. Nevertheless, botanical specimens have been observed and collected also in the province of Loja, confirming the presence of *B. sinuata* in Southern Ecuador [10,11]. According to the World Flora Online (WFO) Plant List, *Baccharis loxensis* Benth. and *Tursenia sinuata* Cass. are synonyms for *B. sinuata*, whereas, in accordance with the Missouri Botanical Garden (Tropicos.org), no synonyms are associated with this taxon [8,9]. On one hand, hundreds of papers have been published about the phytochemistry of the genus *Baccharis* and the biological activities of secondary metabolites from this taxon. From 2005 to 2016, 139 compounds were identified from 27 species, with flavonoids, phenolic acids from the shikimate pathway, and terpenes of all classes as the most common substances [12]. According to the literature, monoterpenes, monoterpenoids, sesquiterpenes, and sesquiterpenoids constitute the volatile fractions of many *Baccharis* spp., converting this botanical genus in a good source of EOs. A lot of these volatile fractions have been submitted to biological assays, affording positive results. Antibacterial, antiulcer, antiprotozoal, antifungal, anti-inflammatory, insect repellent, and sedative capacities, among others, have been demonstrated [12]. If we reduce our interest to EOs, based on SciFinder database, about 180 papers in English have been published so far. Of all these results, only two refer to volatile fractions from Ecuadorian species: *B. obtusifolia* and *B. latifolia*, both from the province of Loja, and characterised for being dominated by limonene as the major compound (28.3% and 33.7%, respectively) [13,14]. On the other hand, no information has been found on the biological activities and phytochemical studies for *B. sinuata* or any of its synonyms, as well as the ethnopharmacological applications. Based on this evidence, the present study represents, to the best of the authors’ knowledge, the first chemical and enantioselective analysis of an EO, distilled from the leaves of *B. sinuata*.

## 2. Results

### 2.1. Qualitative and Quantitative Chemical Analyses

**Figure 2:**
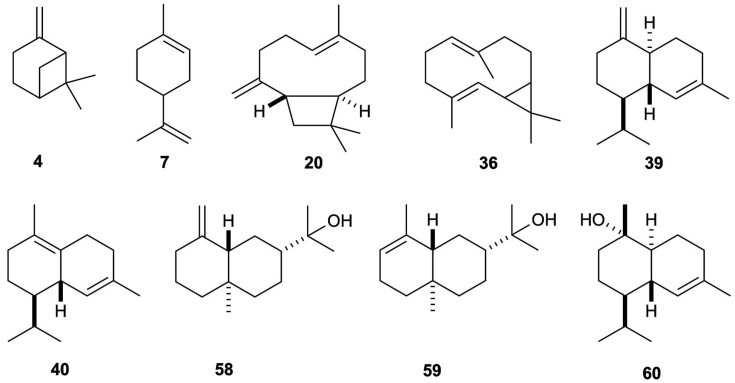
Major components (≥2.0%) of *B. sinuata* leaf EO. The numbers refer to Table 1: β-pinene (**4**), limonene (**7**), (*E*)-β-caryophyllene (**20**), bicyclogermacrene (**36**), γ-cadinene (**39**), δ-cadinene (**40**), β-eudesmol (**58**), α-eudesmol (**59**), α-cadinol (**60**).

**Figure 3:**
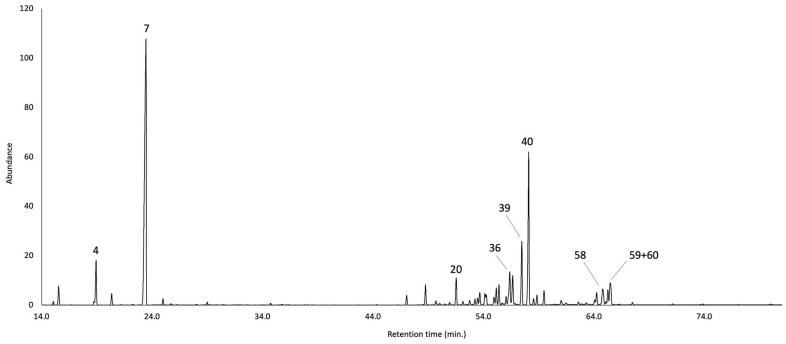
GC-MS profile of *B. sinuata* leaf EO on a 5%-phenyl–methylpolysiloxane stationary phase. The peak numbers refer to the major compounds (≥2.0% as average amount) in Table 1.

**Figure 4:**
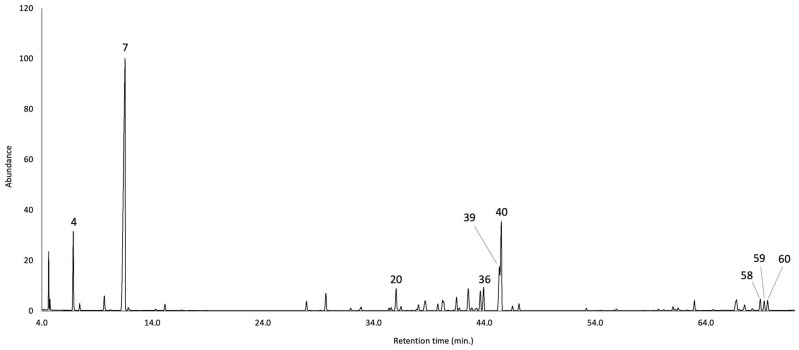
GC-MS profile of *B. sinuata* leaf EO on a polyethylene glycol stationary phase. The peak numbers refer to the major compounds (≥2.0% as average amount) in Table 1.

The leaves of *B. sinuata* produced an EO with a yield of 3.0 ± 0.01% by weight of dry plant material, calculated over three repeated distillations. The EO is characterised by an intense spicy green aroma, somewhat pungent and smoky when concentrated. The qualitative analysis detected 63 compounds, of which 62 were identified. All the detected components were quantified on two different capillary columns whose results, expressed as mean values and standard deviations of six repetitions, are reported in Table 1. Whereas the percent limit of detection by weight has been estimated at 0.05%, the limit of quantification was established at 0.1%. As an average result between the two columns, the quantified constituents corresponded to 97.4% of the whole oil mass, where 49.2% was attributed to monoterpenes and monoterpenoids, and 47.5% was assigned to sesquiterpenes and sesquiterpenoids. The major components of *B. sinuata* EO (≥ 2.0% as average value), sorted by peak number, were β-pinene (4.9%, peak **4**), limonene (39.0%, peak **7**), (*E*)-β-caryophyllene (2.0%, peak **20**), bicyclogermacrene (2.7%, peak **36**), γ-cadinene (4.0%, peak **39**), δ-cadinene (7.3%, peak **40**), β-eudesmol (2.0%, peak **58**), α-eudesmol (3.0%, peak **59**), and α-cadinol (2.0%, peak **60**). The molecular structures of the most abundant compounds are represented in Figure 2, whereas the gas chromatographic (GC) profiles on the two columns are reported in Figure 3 and Figure 4.

### 2.2. Enantioselective Analysis

The enantioselective analysis focused on 10 chiral compounds, which were analysed on two different chiral selectors, depending on their separation capacity toward each enantiomeric pair. As usual, the number of chiral compounds submitted to enantioselective analysis was limited by the commercial availability of enantiomerically pure standards. Among the analysed metabolites, (1*R*,5*R*)-(−)-α-thujene, (1*S*,5*S*)-(−)-α-pinene, and (1*R*,2*S*,6*S*,7*S*,8*S*)-(−)-α-copaene were enantiomerically pure, whereas (*R*)-(+)-limonene presented a 90.0% enantiomeric excess. All the other chiral compounds were scalemic mixtures, with an enantiomeric excess in the range 34.2–85.6%. The complete results of the enantioselective analysis are detailed in Table 2.

## 3. Discussion

The genus *Baccharis* has been widely investigated as a source of Eos, and many of these volatile fractions have been described in the literature for their chemical compositions and biological activities [12,55]. The chemical profiles of *Baccharis* spp. EOs are very diverse, and different species are dominated by different compounds, sometimes in very important amounts. These are the cases of (*E*)-nerolidol and spathulenol, that exceeded 68% and 95%, respectively, in the EOs of *B. tricuneata* var. *ruiziana* and *B. crispa* [56,57]. Other important examples are β-pinene in *B. milleflora* (67.5%), *B. articulata* (30.1%), and *B. stenocephala* (41.3%); carquejyl acetate in *B. genistelloides* (42.8%); bicyclogermacrene (42.4%) and verboccidentafuran (43.9–47.5%) in different sample of *B. punctulata*; and isocaryophyllene (34.3%) in *B. coridifolia* [58,59,60,61,62,63]. Finally, another important group is the one where limonene is the first or second most abundant component, including species such as *B. calvescens* (20.6%, second main compound after spathulenol), *B. uncinella* (88.8%, main compound), *B. trimera* (42.2%, main compound), and *B. milleflora* (28.2%, second main compound after β-pinene) [58]. It is evident that *B. sinuata* belongs to the last group, with limonene (**7**) as the major EO component, reaching 39.0% of the whole oil mass. In particular, the enantioselective analysis showed that 95.0% of limonene was present as its dextrorotatory form, whose biological properties possibly influence the whole EO. According to the literature, (*R*)-(+)-limonene specifically presents a wide variety of biological properties. Among these, it increases parasympathetic activity and lowers the heart rate by olfactory stimulation [64]. Furthermore, antidepressant-like, anticancer (it promotes autophagy and inhibits tumour growth), antidiabetic, and anti-inflammatory properties have been reported [65,66,67,68,69]. Finally, in a study, it prevented mitotic spindle assembly, confirming a potential role as an anticancer product [70].

The second component that, due to its abundance, could significantly affect the biological properties of *B. sinuata* EO is δ-cadinene (7.3%, **40**). As for most sesquiterpenes, due to the unavailability of enantiomerically pure standards, no results could be obtained about δ-cadinene from the enantioselective analysis. Furthermore, as is usual for sesquiterpenes, no clear results are available in the literature for the biological activities of pure compounds. On the other hand, some information is available for EOs where δ-cadinene is a major component. In this respect, the most reported biological activity is the antimicrobial capacity of EOs from *Beilschmiedia madang*, *Kadsura longipedunculata*, and *Xylopia laevigata*, together with volatile fractions from other species of genus *Beilschmiedia* and *Siparuna* [71,72,73,74,75]. Other less investigated biological activities that can be attributed to δ-cadinene are antioxidant, anti-inflammatory, cholinergic, antifungal, anticancer, antityrosinase, trypanocidal, and larvicidal [71,72,73,74].

The third most abundant constituent was γ-cadinene (4.0%, **39**). As for its regioisomer δ-cadinene, compound **39** has not been exhaustively studied, as a pure compound, in terms of its biological activities. However, as usual, some biological properties have been reported for EOs with relatively high contents of this sesquiterpene. This is, for example, the case of the volatile fractions distilled from the rhizomes of *Kaempferia galanga* and from the leaves and aerial parts of *Schinus molle* and *Helichrysum microphyllum* ssp. *tyrrhenicum*. In these EOs, γ-cadinene reached amounts as high as 9.8%, 9.1%, and 6.7%, respectively [76,77,78]. The biological activities that, shared by these three EOs, could be associated to γ-cadinene as a common major compound were the antibacterial and the antitumoral/cytotoxic capacities. Furthermore, *K. galanga* EO also showed many other biological activities, such as antifungal, anti-inflammatory, analgesic, antiviral, antihypertensive, antinociceptive, and antispasmodic, among others [76,77,78]. Comparing these properties with the ones of δ-cadinene, the antibacterial and anticancer capacities are common to both compounds, suggesting a possible synergic effect.

We must consider the results of the enantioselective analysis. In this EO, we can observe that the major enantiomer of sabinene was the one with absolute configuration (*1S,5S*), whereas α-thujene was exclusively constituted by the form with configuration (*1R,5R*). Likewise, (*R*)-(+)-limonene was enantiomerically dominant, whereas (*S*)-(−)-α-terpineol was the enantiomer that presented an enantiomeric excess. This phenomenon, despite not being unusual, is remarkable, because it is well-known that the thujyl cation is the precursor of both sabinene and thujene, which inherit the same asymmetric centres. The same occurs for α-terpineol and limonene, both deriving from the same α-terpinyl cation. In all these cases, the stereogenic centres are defined in precursors, and their configurations should be maintained in each product [79]. For these reasons, an enantiomeric excess in favour of the enantiomers with the same absolute configuration should be expected for the α-thujene/sabinene and limonene/α-terpineol pairs. The fact that this expectation was not met suggests two possible hypotheses: (1) distinct biosynthetic pathways may give rise to different chiral compounds even when they originate from the same precursor; (2) different chiral compounds deriving from a common precursor are produced with the same enantiomeric excess but suffer different enantiospecific post-synthetic transformations that change their enantiomeric distribution. Finally, in addition to these biochemical explanations, the hypothesis of a partial racemisation must be considered, at least for the limonene/α-terpineol pair. According to this hypothesis, during steam distillation, the EO was submitted to a high temperature treatment in an aqueous medium, whose conditions could also be somehow acidic if organic acids are present in plant material. In these conditions, it has been demonstrated that tertiary alcohols, such as α-terpineol, can suffer racemisation, due to the formation of a stable tertiary carbocation at the chiral centre. To a lesser extent, unsaturated alkenes, such as limonene, could also undergo a similar process through protonation [80,81,82]. The latter explanation should remind us that EOs are laboratory products, defined by how they are obtained, and possibly characterised by the presence of sometimes characteristic artifacts (e.g., chamazulene in *Matricaria chamomilla* EO). For these reasons, in EO chemistry, both chemical composition and enantiomeric distribution should not be used to rigorously derive biochemical statements, since EOs do not strictly represent metabolic profiles.

## 4. Materials and Methods

### 4.1. Plant Material

The leaves of *B. sinuata* were collected on 23 August 2024, at an altitude of 2390 m above sea level. The whole plant material was obtained from many shrubs, spread around a central point of coordinates 3°35′10″ S and 79°14′25″ W, within a range of about 200 m. After collection, the leaves were collated to form a unique mean sample, which was dried at 35 °C for 48 h, in a temperature controlled drying room, to better preserve the plant material until distillation. The water content was about 55% of the fresh leaf mass, calculated through the ratio between the fresh and dry plant material. The dry plant material was packaged, in an opaque bag, at about 20 °C until use. The botanical identification was conducted by one of the authors (N.C.), based on original samples that are conserved in the herbarium of the Universidad Técnica Particular de Loja (HUTPL, Loja, Ecuador). Both the collected and reference specimens coincided in their morphological aspects. A botanical voucher of the collected specimens was also deposited in the same herbarium, with code 15458. The present study was carried out in agreement with the Ecuadorian law, by authorisation of the Ministry of Environment, Water, and Ecological Transition of Ecuador (MAATE), with permit code MAATE-DBI-CM-2022-0248.

### 4.2. Essential Oil Distillation and Sample Preparation

The whole dry plant material was divided into three portions of 185.5 g, 150.0 g, and 47.6 g, respectively, all corresponding to the same mean biological sample, which is representative of different shrubs. Each portion was steam-distilled for 4 h in a modified Dean–Stark apparatus, as previously described in the literature, obtaining three replicates of the same EO [83]. Each distilled replicate was dried over anhydrous sodium sulphate, purchased from Merck (Sigma–Aldrich, St. Louis, MO, USA), and stored at −15 °C until use. To be analysed in GC, about 5 mg of each EO replicate was diluted with 1 mL of cyclohexane, containing *n*-nonane as internal standard at the concentration of 0.7 mg/mL. Both the solvent and internal standard were provided by Merck (Sigma–Aldrich, St. Louis, MO, USA). For each distillation, two diluted replicates were prepared, obtaining a total of 6 repetitions, which were directly submitted to all GC analyses.

### 4.3. Qualitative Chemical Analysis (GC-MS)

The qualitative analysis was carried out with a GC instrument, coupled to a mass spectrometer (MS) detector. The GC was a model Trace 1310, whereas the MS was a single quadrupole model ISQ 7000, both from Thermo Fisher Scientific (Waltham, MA, USA). The GC oven was equipped with two columns, coated with stationary phases of different polarity: 5%-phenyl–methyl polysiloxane (TR-5ms, non-polar) and polyethylene glycol (TR-WAX, polar). Both columns were 30 m in length, 0.25 mm in internal diameter, and 0.25 μm in phase thickness, purchased from Thermo Fisher Scientific (Waltham, MA, USA). The carrier gas was 99.9995% purity helium, set at the constant flow of 1 mL/min, and provided by Indura S.A. (Guayaquil, Ecuador). With both columns, the following thermal program was applied: 50 °C for 10 min, followed by an initial linear gradient of 2 °C/min up to 170 °C and a second linear gradient of 10 °C/min up to 230 °C, which was maintained for 20 min. The injector was operated in split mode (40:1) and heated at 230 °C, with 1 μL of sample introduced into the injector at each analysis. The transfer line and the ion source were heated at 250 °C. The ion source was an electron impact device, set at the ionisation energy of 70 eV. The mass analyser was operated in SCAN mode, for detecting ions in the range 40–400 *m*/*z*. The qualitative composition of the EO components was determined by comparing each linear retention index (LRI) and mass spectrum with data from the literature in both columns. Whereas reference LRIs were taken from the literature reported in Table 1, the mass spectra were visually compared with the ones from Adams’ book and the NIST Chemistry WebBook [15,84]. In this process, only compounds whose mass spectrum and LRI in both columns coincided with the MS and chromatographic data from the literature were considered identified. In a few cases, marked with symbol § in Table 1, the identification with LRI only applied to one column, due to lack of bibliographic information for the other stationary phase. The LRIs were calculated according to Van den Dool and Kratz, based on a mixture of *n*-alkanes in the range C_9_-C_23_, which were purchased from Merck (Sigma–Aldrich, St. Louis, MO, USA) [85].

### 4.4. Quantitative Chemical Analysis (GC-FID)

The quantitative analysis was performed with the same GC instrument, columns, thermal program, and general analytical conditions as the qualitative ones. However, the injector was set at a split ratio of 10:1, whereas a flame ionisation detector (FID) was used instead of the mass spectrometer. Hydrogen (purity 99.9995%) from an electrolytic generator (Peak Scientifics, Glasgow, UK) and air (purity 99.9995%) from a cylinder (Indura S.A., Guayaquil, Ecuador) were used to aliment the detector flame, with H_2_ flow set at 30 mL/min and air flow at 300 mL/min. Nitrogen (purity 99.9995%) from a cylinder (Indura S.A., Guayaquil, Ecuador) was used as a makeup gas, with flow 300 mL/min. All the detected compounds were quantified applying a six-point calibration curve for each column, where points were linearly interpolated. Both curves were traced using isopropyl caproate as a quantification standard and *n*-nonane as internal standard, producing a correlation coefficient > 0.995. The six diluted standards for the construction of the calibration curve were prepared weighting 1.7, 2.5, 4.3, 8.6, 16.7, and 34.6 mg of isopropyl caproate, each one spiked with about 7 mg of *n*-nonane (see Supplementary Material), and filling each of them to 10 mL with cyclohexane, as previously described in the literature [86]. Whereas *n*-nonane and cyclohexane were provided by Merck (Sigma–Aldrich, St. Louis, MO, USA), isopropyl caproate was synthesised in the authors’ laboratory and purified to 98.8% (GC-FID purity). Before being applied to the calibration curves, each peak area was multiplied by a relative response factor, calculated as described in the literature, because of its combustion enthalpy [87,88].

### 4.5. Enantioselective Analysis

The enantioselective analysis was conducted by GC-MS, with the same instrument, carrier gas, split mode, and MS parameters described for the qualitative chemical analysis. On the other hand, the injector and transfer line temperatures were set to 220 °C, whereas the gas pressure was maintained constant at 70 kPa, corresponding to an initial flow of about 1 mL/min. Two enantioselective columns were employed, whose stationary phases were based on 2,3-diacetyl-6-*tert*-butyldimethylsilyl-β-cyclodextrin (DAC) and 2,3-diethyl-6-*tert*-butyldimethylsilyl-β-cyclodextrin (DET) as chiral selectors. Both columns, purchased from MEGA s.r.l. (Milan, Italy), were 25 m in length, 0.25 mm in internal diameter, and 0.25 µm in phase thickness. The thermal program was 50 °C for 1 min, followed by a thermal linear gradient of 2 °C/min until 220 °C, which was maintained for 10 min. As previously described, the use of two columns depended on the different separation properties of each enantiomeric pair. All the analysed enantiomers were identified thanks to their mass spectrum and the comparison of the LRIs with the enantiomerically pure standards. Except for α-thujene and due to the commercial unavailability of most of the chiral components of EOs, some enantiomerically pure standards were purchased from Merck (Sigma–Aldrich, St. Louis, MO, USA), whereas others were kindly provided by the University of Turin (Italy). The LRIs for the enantioselective analysis were also calculated according to Van den Dool and Kratz [85], using the same mixture of *n*-alkanes previously described for the qualitative profile.

## 5. Conclusions

The dry leaves of *B. sinuata* produced a high yield EO, characterised by the presence of β-pinene, limonene, (*E*)-β-caryophyllene, bicyclogermacrene, γ-cadinene, δ-cadinene, β-eudesmol, α-eudesmol, and α-cadinol as major compounds (≥3.0% each). This volatile fraction presented a pleasant and distinctive aroma and, according to its chemical composition, it is potentially characterised by a wide range of biological activities. Although *B. sinuata* is a wild species, the high yield (3.0% by weight) makes this EO commercially promising, especially in case of plant domestication. The biological activities of *B. sinuata* leaf EO should be experimentally investigated in further studies, with a special focus on the anticancer, antidiabetic, anti-inflammatory, and antibacterial properties. Moreover, three hypotheses were proposed to explain the presence of chiral constituents that displayed unexpected enantiomeric excesses.

## Figures and Tables

**Table 1:** Qualitative (gas chromatography–mass spectrometry, GC-MS) and quantitative (gas chromatography–flame ionisation detector, GC-FID) chemical composition of *B. sinuata* leaf EO. The analyses were conducted on 5% phenyl–95% methyl polysiloxane, expressed by weight, and 100% polyethylene glycol stationary phases. The major components (≥2.0% as average value) are reported in bold.

N.	Compounds	5% Phenyl Methyl Polysiloxane					Polyethylene Glycol					Average (%)
		LRI		%	σ	Lit.	LRI		%	σ	Lit.	
		Calc.	Ref.				Calc.	Ref.				
1	α-thujene	927	924	0.3	0.09	[15]	1018	1018	0.4	0.12	[16]	**0.4**
2	α-pinene	933	932	1.9	0.47	[15]	1014	1014	1.9	0.40	[17]	**1.9**
3	sabinene	972	969	0.4	0.09	[15]	1112	1112	0.4	0.08	[18]	**0.4**
4	**β-pinene**	974	974	4.9	0.86	[15]	1101	1100	4.9	0.62	[19]	**4.9**
5	myrcene	992	988	1.3	0.25	[15]	1154	1154	1.3	0.19	[20]	**1.3**
6	α-terpinene	1016	1014	0.1	0.01	[15]	1164	1164	0.1	0.02	[21]	**0.1**
7	**limonene**	1030	1024	39.0	6.99	[15]	1190	1190	39.0	4.96	[22]	**39.0**
8	(*E*)-β-ocimene	1049	1044	0.5	0.10	[15]	1245	1245	0.5	0.08	[23]	**0.5**
9	γ-terpinene	1058	1054	0.1	0.02	[15]	1233	1233	0.1	0.01	[24]	**0.1**
10	linalool	1099	1095	0.3	0.02	[15]	1551	1551	0.3	0.03	[25]	**0.3**
11	terpinen-4-ol	1174	1174	0.2	0.04	[15]	1592	1592	0.1	0.04	[26]	**0.2**
12	α-terpineol	1187	1186	0.1	0.02	[15]	-	-	-	-	-	**0.1**
13	*p*-vinyl-guaiacol	1309	1309	0.1	0.01	[15]	-	-	-	-	-	**0.1**
14	α-cubebene	1350	1348	0.6	0.12	[15]	1433	1435	0.6	0.14	[27]	**0.6**
15	α-copaene	1376	1374	1.3	0.16	[15]	1460	1460	1.3	0.21	[28]	**1.3**
16	β-cubebene	1390	1387	0.4	0.01	[15]	1509	1509	0.4	0.04	[29]	**0.4**
17	sibirene	1395	1400	0.1	0.02	[15]	1508	-	0.5	0.17	§	**0.3**
18	methyl eugenol	1402	1403	0.1	0.05	[15]	2016	2016	trace	-	[30]	**0.1**
19	α-gurjunene	1409	1409	0.2	0.02	[15]	1494	1501	0.2	0.03	[31]	**0.2**
20	**(*E*)-β-caryophyllene**	1419	1417	1.9	0.16	[15]	1562	1562	2.0	0.29	[32]	**2.0**
21	β-copaene	1428	1430	0.3	0.03	[15]	1554	1552	0.3	0.04	[33]	**0.3**
22	β-gurjunene	1434	1431	0.1	0.01	[15]	-	-	-	-	-	**0.1**
23	aromadendrene	1438	1439	0.3	0.08	[15]	1607	1606	0.7	0.10	[34]	**0.5**
24	6,9-guaiadiene	1443	1442	0.4	0.03	[15]	1651	-	-	-	§	**0.4**
25	*cis*-muurola-3,5-diene	1446	1448			[15]	-	-	-	-	-	
26	*trans*-muurola-3,5-diene	1450	1451	0.5	0.05	[15]	1595	-	0.3	0.10	§	**0.4**
27	α-humulene	1453	1452	0.9	0.05	[15]	1633	1637	1.6	0.19	[35]	**0.9**
28	9-*epi*-(*E*)-caryophylene	1460	1464	1.6	0.07	[15]	1607	1604	0.3	0.10	[36]	**0.3**
29	*cis*-muurola-4(14),5-diene	1462	1465			[15]	1635	-	o.w. peak 27	-	§	**1.3**
30	*trans*-cadina-1(6),4-diene	1473	1475	0.5	0.05	[15]	1625	-	0.3	0.10	§	**0.4**
31	γ-muurolene	1477	1478	1.1	0.11	[15]	1655	1655	1.1	0.19	[28]	**1.1**
32	germacrene D	1481	1480	1.5	0.08	[15]	1674	1676	2.2	0.14	[35]	**1.9**
33	β-selinene	1485	1489	0.1	0.02	[15]	1679	1678	0.2	0.04	[37]	**0.2**
34	β-*cis*-guaiene	1488	1492			[15]	1659	1651	0.2	0.06	[38]	**0.2**
35	*trans*-muurola-4(14),5-diene	1491	1493	1.1	0.10	[15]	1687	-	0.5	0.09	§	**0.8**
36	**bicyclogermacrene**	1496	1500	3.0	0.13	[15]	1698	1699	2.3	0.12	[35]	**2.7**
37	α-muurolene	1500	1500	1.7	0.29	[15]	1693	1695	1.7	0.21	[39]	**1.7**
38	germacrene A	1504	1508	0.1	0.00	[15]	-	-	-	-	-	**0.1**
39	**γ-cadinene**	1514	1513	4.0	0.10	[15]	1725	1722	11.0	1.11	[40]	**4.0**
40	**δ-cadinene**	1525	1522	7.3	0.41	[15]	1728	1729			[27]	**7.3**
41	*trans*-cadina-1,4-diene	1532	1533	0.4	0.01	[15]	1747	-	0.4	0.04	§	**0.4**
42	α-cadinene	1538	1537	0.6	0.04	[15]	1758	1751	0.6	0.06	[41]	**0.6**
43	elemol	1548	1548	1.5	0.69	[15]	2075	2074	1.5	0.57	[42]	**1.5**
44	palustrol	1566	1567	0.1	0.06	[15]	1899	1903	trace	-	[27]	**0.1**
45	germacrene D-4-ol	1575	1574	1.1	0.78	[15]	2034	2038	0.3	0.08	[43]	**0.7**
46	spathulenol	1577	1577			[15]	2111	2110	0.4	0.26	[44]	**0.4**
47	globulol	1582	1590	0.5	0.35	[15]	2060	2061	0.1	0.04	[45]	**0.3**
48	unidentified (MW = 222)	1590	1590	0.2	0.20	[15]	-	-	-	-	-	**0.2**
49	ledol	1602	1602	0.5	0.29	[15]	2006	2007	0.1	0.02	[46]	**0.3**
50	β-oplopenone	1607	1607	0.2	0.22	[15]	-	-	-	-	-	**0.2**
51	1,10-di-*epi*-cubenol	1614	1618	0.3	0.22	[15]	2038	2037	0.2	0.05	[47]	**0.3**
52	10-*epi*-γ-eudesmol	1618	1622	0.2	0.15	[15]	2080	2084	0.2	0.12	[36]	**0.2**
53	1-*epi*-cubenol	1628	1627	0.6	0.38	[15]	2044	2047	0.3	0.25	[46]	**0.5**
54	γ-eudesmol	1631	1630	2.0	1.04	[15]	2156	2158	3.0	1.42	[48]	**2.0**
55	*epi*-α-cadinol	1641	1638	3.0	1.44	[15]	2158	2158			[49]	**1.2**
56	*epi*-α-muurolol	1642	1640			[15]	2174	2173	1.3	0.62	[50]	**1.3**
57	α-muurolol	1646	1644	0.4	0.24	[15]	2190	2191	0.5	0.27	[51]	**0.5**
58	**β-eudesmol**	1649	1649	2.0	1.30	[15]	2217	2216	2.0	1.36	[52]	**2.0**
59	**α-eudesmol**	1653	1652	5.0	2.80	[15]	2207	2208	3.0	1.33	[48]	**3.0**
60	**α-cadinol**	1654	1652			[15]	2225	2225	2.0	1.12	[53]	**2.0**
61	shyobunol	1689	1688	0.4	0.26	[15]	-	-	-	-	-	**0.4**
62	benzyl benzoate	1769	1759	0.2	0.16	[15]	-	-	-	-	-	**0.2**
63	*n*-docosane	2200	2200	0.3	0.07	[15]	-	-	-	-	-	**0.3**
	monoterpenes			48.5					48.6			48.6 *
	oxygenated monoterpenoids			0.6					0.4			0.6 *
	sesquiterpenes			30.0					28.7			30.4 *
	oxygenated sesquiterpenoids			18.0					14.9			17.1 *
	others			0.7					-			0.7 *
	total			97.8					92.6			97.4 *

N. = progressive number; Calc. = calculated linear retention index; Ref. = reference linear retention index; % = percent by weight of EO; σ = standard deviation; Lit. = reference literature; § = identification by MS only; trace = < 0.1%; o.w. = overlapped with; Average = mean amount between the two columns. If in one column the component is trace, undetected, or the sum of two peaks, only the value of the other column is reported. * These results are not calculated as mean amounts between the columns but as sums of the average values.

**Table 2:** Enantioselective analysis of some chiral terpenes from *B. sinuata* leaf EO.

Chiral Selector	Ion Integration (*m/z*)	LRI	Enantiomer	Distribution (%)	e.e. (%)
DET	TIC	913	(*1S,5S*)-(+)-α-thujene *	-	100.0
DET	TIC	917	(*1R,5R*)-(−)-α-thujene *	100.0	
DAC	TIC	914	(*1S,5S*)-(−)-α-pinene	100.0	100.0
DAC	TIC	916	(*1R,5R*)-(+)-α-pinene	-	
DET	TIC	949	(*1R,5R*)-(+)-β-pinene	12.0	76.0
DET	TIC	958	(*1S,5S*)-(−)-β-pinene	88.0	
DET	TIC	977	(*1R,5R*)-(+)-sabinene	12.4	75.2
DET	TIC	991	(*1S,5S*)-(−)-sabinene	87.6	
DET	TIC	1056	(*S*)-(−)-limonene	5.0	90.0
DET	TIC	1067	(*R*)-(+)-limonene	95.0	
DET	TIC	1181	(*R*)-(−)-linalool	81.7	63.4
DET	TIC	1194	(*S*)-(+)-linalool	18.3	
DAC	TIC	1293	(*R*)-(−)-terpinen-4-ol	76.2	52.4
DAC	TIC	1298	(*S*)-(+)-terpinen-4-ol	23.8	
DET	59	1301	(*S*)-(−)-α-terpineol	67.1	34.2
DET	59	1313	(*R*)-(+)-α-terpineol	32.9	
DET	TIC	1322	(*1R,2S,6S,7S,8S*)-(−)-α-copaene	100.0	100.0
DET	TIC	1324	(*1S,2R,6R,7R,8R*)-(+)-α-copaene	-	
DET	161	1461	(*R*)-(+)-germacrene D	7.2	85.6
DET	161	1467	(*S*)-(−)-germacrene D	92.8	

DAC = 2,3-diacetyl-6-*tert*-butyldimethylsilyl-β-cyclodextrin; DET = 2,3-diethyl-6-*tert*-butyldimethylsilyl-β-cyclodextrin; TIC = total ion current; LRI = calculated linear retention index; * tentative attribution based on [54]; e.e. = enantiomeric excess.

## Data Availability

The datasets presented in this article are not readily available because they are part of an ongoing study. Requests to access the datasets should be directed to the corresponding author.

## Supplementary Materials

The following supporting information can be downloaded at https://www.mdpi.com/article/10.3390/plants14193110/s1, Figure S1: Calibration curves for the GC-FID analyses of *B. sinuata* EO on a non-polar and polar stationary phase; Table S1: Origin and purity grade of the enantiomerically pure standards.
