# Supplementary material for: A Bioeconomically Valuable Essential Oil from Baccharis sinuata Kunth in Southern Ecuador: Chemical Composition and Enantiomeric Profile
Source: Plants (Basel). 2025 Oct 9;14(19):3110. doi: 10.3390/plants14193110 (PMC12526644; doi:10.3390/plants14193110)
Supplement: Supplementary file 1 [file plants-14-03110-s001.zip › plants-3887205-supplementary.pdf]

SUPPLEMENTARY MATERIAL

A Bioeconomically Valuable Essential Oil from *Baccharis sinuata* Kunth in Southern Ecuador: Chemical Composition and Enantiomeric Profile

Gianluca Gilardoni [1], Bryan Flores [2], Nixon Cumbicus [3] and Omar Malagón [1,*]

[1] Departamento de Química, Universidad Técnica Particular de Loja (UTPL), Calle Paris s/n y Praga, Loja 110107, Ecuador; ggilardoni@utpl.edu.ec or gianluca.gilardoni@gmail.com

[2] Carrera de Bioquímica y Farmacia, Universidad Técnica Particular de Loja (UTPL), Calle Paris s/n y Praga, Loja 110107, Ecuador; beflores6@utpl.edu.ec

[3] Departamento de Ciencias Biológicas y Agropecuarias, Universidad Técnica Particular de Loja (UTPL), Calle Paris s/n y Praga, Loja 110107, Ecuador; nlcumbicus@utpl.edu.ec

* Correspondence: omalagon@utpl.edu.ec

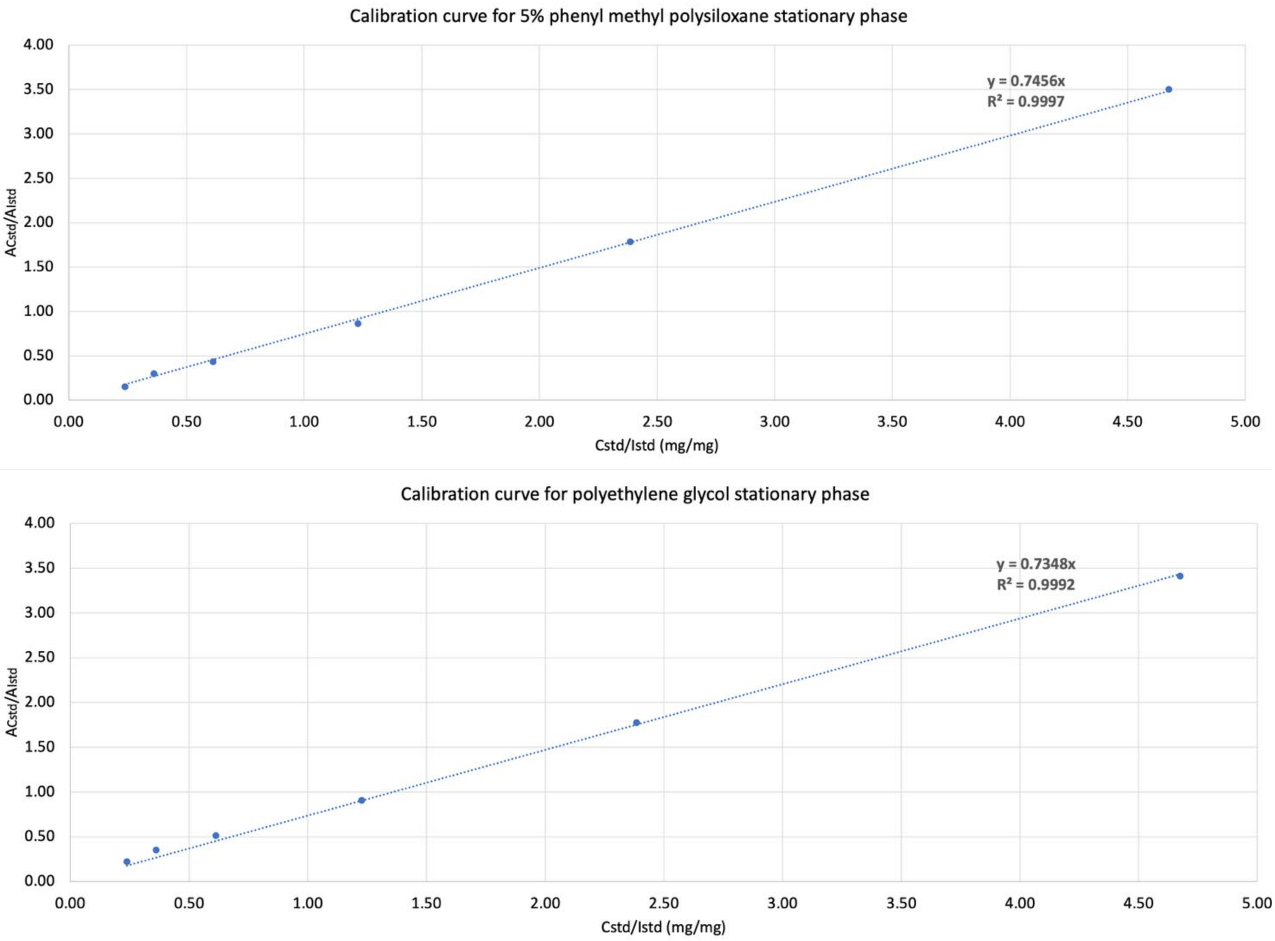

Figure S1. Calibration curves for the GC-FID analyses of *B. sinuata* EO on a non-polar and polar stationary phase. C_{std}: calibration standard (isopropyl caproate); I_{std}: Internal standard (*n*-nonane); A_{Cstd}: Integration area of the internal standard; A_{Cstd}: Integration area of the calibration standard.

Table S1. Origin and purity grade of the enantiomerically pure standards.

Standard	Provider	Product code	Grade	Note
(*1S,5S*)-(–)-α-pinene	Merck	80599	analytical standard	
(*1R,5R*)-(+)-α-pinene	Merck	80605	analytical standard	
(*1R,5R*)-(+)-β-pinene	Merck	80607	analytical standard	
(*1S,5S*)-(–)-β-pinene	Merck	80609	analytical standard	
(*1R,5R*)-(+)-sabinene	Kindly from University of Turin	unavailable	unavailable	
(*1S,5S*)-(–)-sabinene	Kindly from University of Turin	unavailable	unavailable	
(*S*)-(–)-limonene	Merck	62128	analytical standard	
(*R*)-(+)-limonene	Merck	62118	analytical standard	
(*R*)-(–)-linalool	Merck	74856	analytical standard	
(*S*)-(+)-linalool	Merck	51782	analytical standard	Mixture of isomers
(*R*)-(–)-terpinen-4-ol	Merck	49598	analytical standard	
(*S*)-(+)-terpinen-4-ol	Merck	86477	analytical standard	
(*S*)-(−)-α-terpineol	Merck	4899	analytical standard	
(*R*)-(+)-α-terpineol	Merck	77663	analytical standard	Mixture of isomers
(*1R,2S,6S,7S,8S*)-(–)-α-copaene	Kindly from University of Turin	unavailable	unavailable	
(*1S,2R,6R,7R,8R*)-(+)-α-copaene	Kindly from University of Turin	unavailable	unavailable	
(*R*)-(+)-germacrene D	Kindly from University of Turin	unavailable	unavailable	
(*S*)-(–)-germacrene D	Kindly from University of Turin	unavailable	unavailable	
